# Analysis of Breast Cancer Cell Invasion Using an Organotypic Culture System

**DOI:** 10.1007/978-1-4939-7021-6_15

**Published:** 2017-02-20

**Authors:** Romana E. Ranftl, Fernando Calvo

**Affiliations:** Tumour Microenvironment Team, Division of Cancer Biology, Institute of Cancer Research, 237 Fulham Road, London, SW2 6JB UK

**Keywords:** Organotypic, Invasion, Metastasis, Breast cancer, Cancer-associated fibroblasts

## Abstract

Metastasis is the main cause of cancer patient mortality. Local tumor Invasion is a key step in metastatic dissemination whereby cancer cells dislodge from primary tumors, migrate through the peritumoral stroma and reach the circulation. This is a highly dynamic process occurring in three dimensions that involves interactions between tumor, stromal cells, and the extracellular matrix. Here we describe the organotypic culture system and its utility to study breast cancer3D culture model cell Invasion induced by cancer-associated fibroblasts. This is a three-dimensional model that reproduces the biochemical and physiological properties of real tissue and allows for investigating the molecular and cellular mechanisms involving tumor and its microenvironment, and their contribution to cancer cell Invasion. This system provides a robust, accurate, and reproducible method for measuring cancer cell Invasion and represents a valuable tool to improve the mechanistic understanding of the initial steps in Metastasis.

## Introduction

Metastatic dissemination Breast cancer Breast cancer cells Invasionis the major clinical complication in most types of cancer and the cause of 90% of cancer-related deaths [1]. Invasion of cancer cells into the peritumoral stroma is a key step in Metastasis [2, 3]. Thus, understanding the mechanisms regulating the invasive abilities of cancer cells is imperative to inform the development of therapeutic modalities to minimize cancer dissemination and improve patient survival [4]. Local cancer cell Invasion is a multifunctional and dynamic process occurring in three dimensions that is actively modulated by the peritumoral stroma [5–7]. Cancer-associated Fibroblasts) are particularly relevant as they can produce soluble factors that promote cancer cell Invasion [8]. In addition, CAF-dependent Matrix remodeling via focalized Proteolysis activity [9] or Actomyosin-dependent force generation [10, 11] can lead to the formation of tracks through the extracellular matrix (ECMextracellular matrix (ECM)) that enable subsequent cancer cell Invasion. Accordingly, three-dimensional (3D) models of cancer 3D culture modelcell Invasion that incorporate stromal components such as fibroblasts and physiologically relevant ECMs recapitulate more closely the in vivo situation. These models provide a platform for investigating the complex interactions between tumor and its microenvironment that are more likely to lead to novel insights of clinical relevance.

**Fig. 1:**
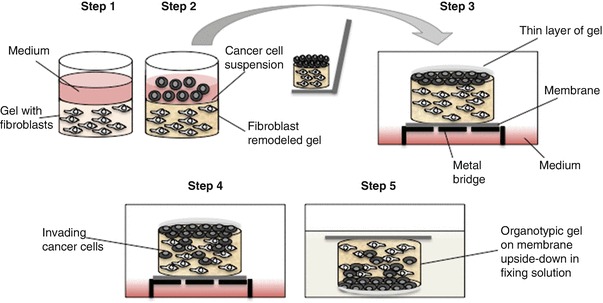
Schematic representation of the workflow of an organotypic Invasion assay. Step 1: Embed fibroblasts in gel and seed in 24-well dish. Step 2: Cancer cells are seeded in a single-cell suspension on top of the gel. Step 3: Once the cells have adhered, remove the medium and lift the remodeled gel onto gel-coated Nylon filter on a metal bridge. Coat the cancer cells with a thin layer of gel. Step 4: Feed with complete medium up to the Nylon filter. Incubate at 37 °C, 5% CO_2_ for 5 days to allow for cancer cell Invasion. Step 5: Terminate assay by fixing organotypic gels. Process gels for H&E staining

Here, we describe a 3D organotypic Invasion assay3D organotypic invasion assay adapted for the robust and accurate Assessment of Breast cancer 3D culture model cell Invasion induced by CAFsFibroblasts [11–13]. This approach was originally developed as a 3D Coculture model by Fusenig and colleagues [14], and further developed by other groups to study squamous cell Carcinoma Invasion [10, 15]. Briefly, CAFsFibroblasts are embedded in a dense gel composed of fibrillar collagen I and basement membrane matrix (termed Matrigel ^®^, Cultrex^®^, or Engelbroth-Holm-Swarm matrix), which contains laminins, collagen IV, Proteoglycans, and a broad spectrum of Growth factors (*see* Fig. 1). A thin layer of gel is used to cover the breast cancer3D culture model cells seeded on the surface of the CAF-containing gel to mimic the physiological condition of breast tissue. Gels are subsequently laid on a grid and maintained partially immersed in cell medium. Organotypic gels are then fixed and processed by standard histopathological procedures followed by quantitative and qualitative analysis of breast cancer3D culture model cell Invasion using standard image processing and analysis software.

## Materials

### Tissue Culture

1. Cancer-associated Fibroblasts):
  - For the mouse model: CAFsFibroblasts from breast carcinomas from the FVB/n MMTV-PyMT murine model [11, 16].
  - For the human model: CAFsFibroblasts from a resection of a human breast Carcinoma.
2. Normal fibroblasts (NFs):
  - For the mouse model: NFs from mammary glandsMammary gland (MG) of FVB/n wild-type siblings [11, 16].
  - For the human model: NFs from a resection of a reduction mammoplasty.
3. Breast cancer cells:
  - For the mouse model: 410.4 or 4T1 cells (ATTC^®^-CRL-2539™) (*see* **Note** **1**).
  - For the human model: MDA-MB-231 (ATCC^®^-HTB-26) (*see* **Note** **2**).
4. Fibroblast culture medium: 10% fetal bovine serum (FBS), 1× GlutaMax™ (Gibco^®^) and 1× insulin–transferrin–selenium (ITS, Gibco^®^) in Dulbecco’s modified Eagle’s medium (DMEM). Store at 4 °C. Warm up before use.
5. Cancer cell culture medium: 10% FBS and 1× GlutaMax™ in DMEM (*see* **Note** **3**). Store at 4 °C. Warm up before use.
6. Sterile phosphate buffered saline (PBS): 3.2 mM Na_2_HPO_4_, 0.5 mM KH_2_PO_4_, 1.3 mM KCl, 135 mM NaCl, pH 7.4. Warm up before use.
7. Sterile 0.05% trypsin–0.02% EDTA. Store at 4 °C. Warm up before use.
8. If a Killing Assay is performed: selective compounds such as puromycin (puromycin dihydrochloride from *Streptomyces alboniger*, Sigma) (*see* **Note** **4**).

### Gel Preparation

1. 5× DMEM: 5% αDMEM powder (Gibco^®^), 2% NaHCO_3_ (0.24 M NaHCO_3_), 0.1 M Hepes pH 7.5. Store at 4 °C (*see* **Note** **5**).
2. Fibroblast culture medium.
3. FBS.
4. Rat-tail collagen type I, high concentration (BD Biosciences). Store at 4 °C (*see* **Note** **6**).
5. Matrigel ^®^ (BD Biosciences). Store at −80 °C in 1 mL aliquots (*see* **Note** **7**).

### Gel Manipulation

1. Nylon NET filters 120 μm (Merck Millipore).
2. Sterile stainless steel metal bridges with a grid size of 2–3 mm. Approximate dimensions: 21 × 21 × 5 mm (Fig. 2a).

**Fig. 2:**
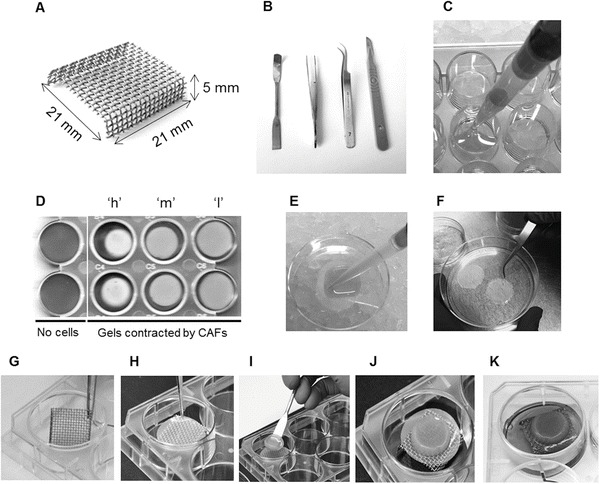
Gel preparation and processing steps. (**a**) Metal bridges with approximate dimensions indicated. (**b**) Useful tools for handling the organotypic cultures, Nylon filters and metal bridges. (**c**) Cell-free or fibroblast-containing gels are plated in a 24-well plate on ice. (**d**) The ability of fibroblasts to contract the collagen:Matrigel gel (a measure of their Matrix remodeling capacity) can be documented prior to further processing. Images show duplicate samples of organotypic cultures free of cells and with different human CAFsFibroblasts that have low (“l”), medium (“m”), and high (“h”) contractility, as indicated. (**e**, **f**) Nylon filters are soaked in gel (**e**) and then separated in a culture dish for setting and fixing (**f**). (**g**–**k**) Setting up the organotypic gel on the metal bridges. Sterile bridges are placed in a 6-well plate (**g**) and covered by a Nylon filter (**h**). The organotypic cultures are lifted from the 24-well plate and placed over the filter:bridge using a spatula (**i**); a thin gel layer is added on the top to cover the cancer cell monolayer (**j**); and complete medium is added to the 6-well dish until soaking the Nylon filter underneath the organotypic gel (**k**)
3. Sterile forceps and spatula (Fig. 2b).
4. Sterile PBS.
5. Filter fixing solution: 4% paraformaldehyde (PFA) and 0.25% glutaraldehyde in PBS.

### Post Processing and Analysis

1. Gel fixing solution: 4% PFA and 1% glutaraldehyde in PBS.
2. Scalpel and forceps (Fig. 2b).
3. 70% ethanol.
4. Standard materials for paraffin embedding and hematoxylin and eosin (H&E) staining.
5. Bright field microscope with 20× (or 10×) objective and camera.
6. Image Image software (Image J—http://imagej.nih.gov/ij—or similar).

## Methods

This protocol is an adapted version of a previously described method for SCC12 Carcinoma cells [10] (*see* Fig. 1 for a schematic representation). We have optimized the Organotypic invasion assay Invasionfor murine and human breast cancer3D culture model cells (410.4/4T1 and MDA-MB-231) in combination with murine or human fibroblasts, respectively (*see* **Note** **8**). On a general basis, cancer cells are not invasive in this setting and rely on CAF activities to invade. It is recommended to test the behavior of alternative cancer cell types in the assay by plating them on top of a fibroblast-free gel matrix. Heterogeneity is also observed in CAFsFibroblasts in terms of their ability to contract and remodel gel matrices. The amount of fibroblasts and cancer cells to be used, as well as the length of the protocol, need to be optimized if alternative models are used (*see* **Note** **9**).

### Gel Preparation

All components of the gel mix must be kept on ice. Here we describe the protocol for 1 mL gel mix containing fibroblasts or without fibroblasts. This is the volume required for one sample in one well of a 24-well plate. The total volume of gel mix needs to be scaled up according to sample size. Generally, it is recommended to prepare gel in excess to avoid pipetting errors due to the viscous nature of the components.

1. Prechill all components for the gel preparation on ice.
2. Mix 100 μL FBS, 80 μL 5× DMEM, and 120 μL of fibroblast culture medium.
3. Add 200 μL of Matrigel ^®^ and 400 μL of collagen I (*see* **Notes** **10** and **11**). Keep the mixture on ice (*see* **Note** **12**).
4. Trypsinize Fibroblasts) from a monolayer to a single cell suspension. Count the cells, centrifuge (400 × *g* for 5 min), and resuspend the pellet at a concentration of 10^7^ cells/mL in fibroblast culture medium (*see* **Notes** **13** and **14**).
5. Avoiding bubble formation, add 100 μL of the cell suspension to the gel mix. If a gel without fibroblasts is required, add 100 μL of fibroblast culture medium instead of cell suspension (*see* **Note** **15**).
6. Add 900 μL of the mixture in a 24 well-plate well (Fig. 2c) (*see* **Note** **16**).
7. Place the plate at 37 °C and 5% CO_2_ for 1 h.
8. When the gel is set, add 1 mL of appropriate medium on top and incubate overnight at 37 °C and 5% CO_2_ (*see* **Note** **17**).

### Breast Cancer Cell Preparation

Depending on the number and Matrix-remodeling ability of the fibroblasts, some of the gels may have already contracted at this point. The plate can be scanned to document this, as gel contraction can serve as an additional read-out of CAF function (Fig. 2d).

1. Trypsinize a monolayer of breast Carcinoma cells (4T1, 410.4 or MBA-MD-231), count the cells and prepare a single cell suspension at 5 × 10^6^ cells/mL in cancer cell culture medium.
2. Aspirate the medium carefully from the 24-well plate containing the gels.
3. Apply 100 μL of the cancer cell suspension on the top of each of the gels (*see* **Notes** **18** and **19**).
4. Incubate at 37 °C and 5% CO_2_. Leave the cells to adhere to the matrix for 6–8 h.

### Coating of Nylon Filters

Given the softness of the gels, direct contact with the metal can damage the gels. Before placing the gels on the bridges, Nylon filters have to be prepared and placed in-between the gels and the metal grid.

1. Prepare the adequate number of Nylon filters (1 per organotypic condition) with a sterile forceps on top of each other in a culture dish.
2. Prepare 1 mL of gel without fibroblasts according to the recipe described in Subheading 3.1.
3. Coat the Nylon filters using 1 mL of cell-free gel (Fig. 2e).
4. Separate the coated filters in the culture dish (Fig. 2f).
5. Incubate the coated Nylon filters at 37 °C for 1 h.
6. Fix the coated nylon, using filter fixing solution for at least 2 h at room temperature (RT) or overnight at 4 °C.

### Lifting the Gel and Covering the Cancer Cells

The gels are placed on the metal bridges and medium is fed from underneath to impose a direction of Invasion. The breast cancer3D culture model cell layer is covered with a thin layer of gel for improved simulation of in vivo conditions (*see* **Note** **20**).

1. Wash the gel-coated Nylon filters with sterile PBS for 10 min (×3) to remove all traces of fixing solution.
2. Add cancer cell culture medium to the filters and incubate for 30 min at 37 °C.
3. Place the sterile metal grid bridges in a 6-well plate with sterile forceps (Fig. 2g).
4. Add a coated, washed and medium-adapted Nylon filter on top of each metal bridge with sterile forceps (Fig. 2h).
5. Prepare an appropriate amount of cell-free gel according to the recipe in Subheading 3.1 and keep the mixture on ice (100 μL per organotypic gel).
6. Remove the medium on the top of the gels carefully in order not to disturb the organotypic cultures.
7. Lift the gels with a sterile spatula from the 24-well and place it on a coated filter on top of a metal bridge. The cancer cell layer must be facing up (Fig. 2i, j).
8. Add medium under the bridge until it is in contact with the Nylon filter. Avoid air bubbles between the interphase medium/Nylon filter (Fig. 2k).
9. Add 100 μL of the cell-free gel mixture on top of the lifted gels and spread over the surface.
10. Incubate the culture for 5 days (37 °C, 5% CO_2_) and change the medium daily (*see* **Note** **21**).

### Gel Fixing and Processing

1. After culturing for 5 days (*see* **Note** **9**), remove the medium from the plate and wash with PBS or transfer the organotypic cultures to a new 6-well plate by lifting the filter and gel. Turn the gel upside-down for fixing (cancer cell layer on the bottom, Fig. 1) (*see* **Note** **22**).
2. Fix the gels in 2 mL of gel fixing solution at 4 °C overnight.
3. Next day, wash the gels with PBS (2 mL) for 10 min (×3).
4. Using the forceps and the scalpel cut the gel in two halves and store one of them in 70% ethanol at 4 °C (*see* **Note** **23**).
5. Embed the other half of the gel in paraffin blocks and perform standard H&E staining on sections (*see* **Notes** **24** and **25**).

### Data Analysis

1. Take 5–7 pictures per gel using a bright field microscope (20×/10× magnification) (*see* **Note** **26** and Fig. 3a–e).

**Fig. 3:**
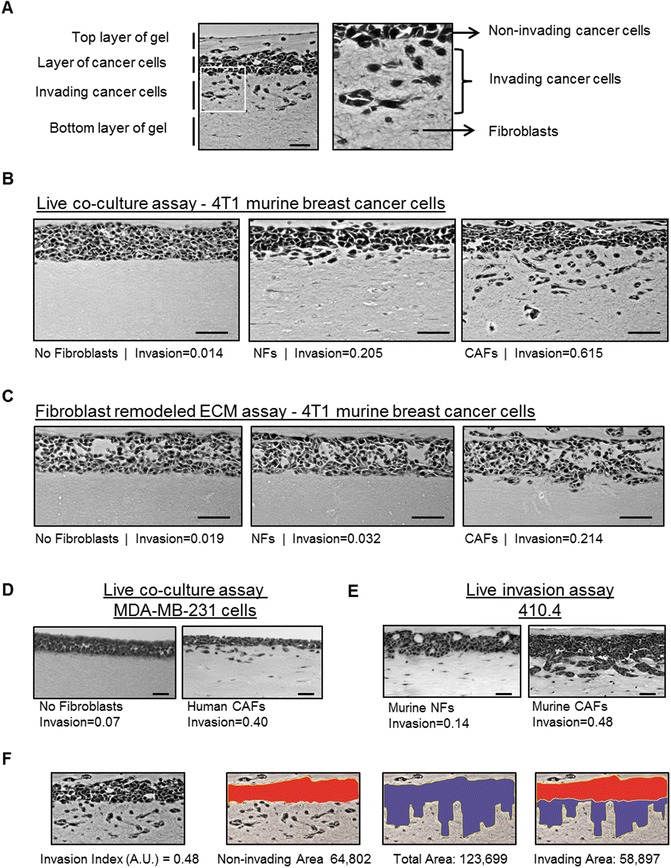
Analysis of cancer cell Invasion using Carcinoma cells in an organotypic Invasion assay. (**a**) Representative image of H&E staining of an organotypic Invasion assay of 4T1 cells cultured in the presence of CAFsFibroblasts. The different parts of the organotypic gel are indicated. *Right panel* is a zoom up region that shows both collective and single cell Invasion of 4T1 cells. Fibroblasts and ECMextracellular matrix (ECM) fibers Invasionare also observed. (**b**) Representative images of H&E staining of Organotypic invasion assays of 4T1 murine breast cancer3D culture model cells cultured in the absence and presence of either murine normal Fibroblasts. Invasion indexes of 4T1 cells for eachFig. 3 (continued) experimental setup are also indicated. Scale bar, 50 μm. (**c**) Representative images of H&E staining of “killing” organotypic Invasion assays of 4T1 murine breast cancer3D culture model cells cultured in gels previously remodeled by murine normal Fibroblasts or mock-remodeled (no fibroblasts). Invasion indexes of 4T1 cells for each experimental set-up are also indicated. Scale bar, 50 μm. (**d**) Representative images of H&E staining of Organotypic invasion assays Invasionof MDA-MB-231 human breastMDA-MB-231, human breast cancer cells cancer3D culture model cells cultured in the absence and presence of human breast cancer3D culture model CAFsFibroblasts. The Invasion index is indicated. Scale bar, 50 μm. (**e**) Representative images of H&E staining of Organotypic invasion assays of 410.4 murine breast cancer3D culture model cells with murine NFs and CAFsFibroblasts. Note that 410.4 cells present a “collective” mode of Invasion. Scale bar, 50 μm. (**f**) Exemplars for image analysis of a representative image of H&E staining of an organotypic Invasion assay of 4T1 cells cultured in the presence of CAFsFibroblasts (shown in panel **a**). First, the non-invaded area (*red*) and the total area (*blue*) are measured using Image J (http://imagej.nih.gov/ij/). The invading area is then calculated by subtracting the non-invading area from the total area. The Invasion index is the ratio of invaded area vs. total area. Invasion index and areas are also indicated
2. Measure the area of noninvasive cancer cells and the total area of cancer cells (noninvasive and invasive) using Image J or a similar software (Fig. 3f).
3. The Invasion Index is calculated by dividing the invading area (total area − invading area) by the total area (*see* **Note** **27**).

### Killing Assay

CAFsFibroblasts have been shown to form tracks in the ECMextracellular matrix (ECM), which has been associated with increased invasiveness of cancer cells [10]. However, CAFsFibroblasts can also produce soluble factors that can promote cancer cell Invasion [8]. The basic Organotypic invasion assay Invasiondescribed above does not allow discriminating between these two abilities.

In order to specifically study the capacity of CAFsFibroblasts to form tracks in the gel that allow subsequent cancer cell Invasion, as well as the potential of cancer cells to invade a gel previously remodeled by CAFsFibroblasts, a variation of the basic organotypic culture assay can be performed: the “killing” assay\“killing\” assay. Fibroblasts are seeded as described earlier and are allowed to remodel the gel matrix for 5 days before they are removed. When cancer cells are seeded on top of the gel, they will invade into the tracks formed by the fibroblasts (Fig. 3c). This modified version also allows for discriminating the selective effect of chemical compounds on fibroblasts/cancer cells as both compartments are never cultured together.

1. Follow the protocol as described in Subheading 3.1 (**steps 1**–**8**). Once the gel is set, add 1 mL of fibroblast culture medium on top.
2. Incubate at 37 °C, 5% CO_2_ for 5 days. Change the medium daily (*see* **Notes** **17** and **28**).
3. Fibroblast removal (“killing” step): Remove the medium from the gels and add 1 mL of fibroblast culture medium containing an appropriate selection compound (e.g., 10 μg/mL puromycin) (*see* **Note** **29**).
4. Incubate at 37 °C, 5% CO_2_ for 48 h.
5. Wash the gels for 1 h in fibroblast culture medium (×3). Incubate at 37 °C, 5% CO_2_ overnight in fibroblast culture medium to remove all traces of selection compound (*see* **Note** **30**).
6. Continue with the procedure as described in Subheadings 3.2–3.6.

## Notes

1. 4T1 cells are a metastatic subpopulation of the original 410.4 murine breast cancerBreast cancer cells 3D culture model cell line [17].
2. For perturbation studies (i.e., RNAi), NFs/CAFsFibroblasts or cancer cells need to be modified before starting the protocol.
3. ATCC and other studies may suggest different media for culturing the breast cancer3D culture model cell lines. However, we recommend using DMEM as the organotypic gels are based on it and it does not affect cell behavior or viability.
4. For puromycin, make aliquots of 10 mg/mL and store at −20 °C.
5. Preparation of 5× αDMEM (250 mL): dissolve 12.5 g DMEM powder (Gibco^®^) and 5 g NaHCO_3_ in 50 mL of sterile MilliQ water. Add 25 mL of sterile 1 M Hepes pH 7.5 and sterile MilliQ water up to 250 mL. Mix and filter the solution using a 0.2 mm filter in sterile conditions. Aliquot in 50 mL and 10 mL aliquots and store at −20 °C. Thaw fresh aliquots for gel preparation and do not store them longer than 30 days at 4 °C.
6. Rat tail collagen type I, high concentration is supplied as a liquid in 0.02 N acetic acid with a concentration range of 8–11 mg/mL. Note the actual concentration of each particular batch when calculating the volumes to be used during gel preparation (*see* **Note** **10**).
7. Matrigel ^®^ is a viscous substance at 4 °C that solidifies at RT. Store Matrigel^®^ in sterile 1 mL aliquots at −80 °C and thaw the amount required for an experiment the night before at 4 °C. Note the actual concentration of each particular batch, as it will be required to calculate the volumes for gel preparation (*see* **Note** **10**). As an alternative to Matrigel^®^ we have successfully used Engelbroth-Holm-Swarm matrix from Sigma-Aldrich in our organotypics.
8. We have used both murine and human CAFsFibroblasts in this assay. In our experience, murine CAFsFibroblasts require higher cell numbers than human CAFsFibroblasts, as the latter tend to be larger in size and have higher ECMextracellular matrix (ECM) remodeling abilities (*see* **Note** **13**). For comparative studies, make sure an equal amount of NFs/CAFsFibroblasts are used.
9. To optimize the approach, it is recommended to empirically determine the effect of two variables: (1) the amount of NFs/CAFsFibroblasts to be embedded on the gel matrix; and (2) the length of the protocol (i.e., how long the fibroblasts are allowed to remodel the gels and/or the cancer cells are allowed to invade).
10. The final concentrations of collagen and Matrigel ^®^ in the gel are set to 4 mg/mL and 2 mg/mL, respectively. The recipe was optimized to have almost physiological concentrations of collagen I and Matrigel^®^. In order to prepare reproducible gel solutions the volumes of the components have to be adapted accordingly, depending on the stock concentrations of Matrigel^®^ and collagen I (*see* **Notes** **6** and **7**). For this protocol we base our volume calculations on stock concentrations of 10 mg/mL for both collagen I and Matrigel^®^. The volume of 5× DMEM can be adapted if larger volumes of the acidic collagen are added to retain optimal buffering.
11. Matrigel ^®^ and collagen I are very viscous and need to be pipetted with care. Avoid bubble formation (as bubbles can be detrimental to the quality of the gel) and retention of gel in the tips (as they may affect the final concentration).
12. Avoid leaving the gel mixture on ice for long periods of time as it may affect its properties.
13. For human NFs/CAFsFibroblasts, resuspend the pellet to a concentration of 2.5 × 10^6^ (*see* **Note** **8**).
14. To reduce the activating effect of FBS in Fibroblasts can be cultured for 5–7 days in fibroblast culture medium supplemented with 0.5% FBS (instead of 10% FBS) prior to the use in the organotypic Invasion assay.
15. Cell-free gel will also be used for the Nylon filter coating procedure (Subheading 3.3) and for covering the cancer cell layer on the top of the organotypic gel (Subheading 3.3).
16. Do not plate the total volume of the mix in order to avoid pipetting errors as the gel is very viscous and sticks to pipette tips. Gels can set at RT; these gels will not have the exact same properties as the ones set at 37 °C. We recommend keeping the 24-well plate on ice while adding the gel, especially when a larger number of samples are handled. This will allow the gel in all wells to set at the same time.
17. At this step, appropriate Growth factors, cytokines or drugs can be added to the medium. Cell medium will buffer some of the acidity of the gels and may turn orange. For optimal results, replace with fresh medium after 2–3 h.
18. Seeding 100 μL of cell suspension on the top of highly contracted gels can be problematic. If needed, in these particular cases the empty space in the well can be covered up with fresh cell-free gel; this will allow cancer cells to be seeded on larger volumes (1 mL of 5 × 10^5^ cell/mL suspension). However, the new gel only attaches loosely to the original gel and can sometimes rip off during the lifting process.
19. If gels have not contracted at all, cancer cells can also be applied in a volume of 1 mL per well (at 5 × 10^5^ cell/mL).
20. Alternatively, depending on the type of Carcinoma cells used in the assay, the cells can also be grown in an air–cells–liquid interface. The cells are prepared and added on top of the fibroblast-containing organotypic culture as described in Subheading 3.2. However, the cells will not be covered with a layer of gel.
21. At this step, appropriate Growth factors, cytokines or drugs can be added to the medium, bearing in mind that they will affect both compartments (i.e., fibroblasts and cancer cells). The gels will become flatter during the incubation on the metal bridges.
22. Alternatively, gels can be snap-frozen for immunofluorescence analysis. Gels are placed in a plastic cuvette, covered by OCT buffer (Tissue-Tek) and immersed in liquid nitrogen. Store at −80 °C until further processing (*see* **Note** **25**).
23. Keep the second half as backup until good H&E staining is obtained or use it to perform additional analysis (*see* **Notes** **22**, **24** and **25**).
24. To allow for Invasion analysis, gels must be embedded in paraffin in the correct orientation. Tissue sections need to be obtained from the cut side of the organotypic gel and not on the top or bottom sides. Sectioning the gel within the paraffin block can be challenging as the noninvasive cell layer can easily come off during processing. We recommend very slow trimming of the paraffin block at the first 500 μm to level the gel. Generate two to three cuts (50 μm apart) per organotypic gel per slide for analysis.
25. As an alternative to H&E staining, gels can also be processed for Immunohistochemistry or immunofluorescence detection of specific cancer cell or CAF markers according to standard protocols (*see* **Note** **22**). This will ascertain the cell identity of the invading cells. Alternatively, fluorescence-labeled cells can be used in the Organotypic invasion assay Invasionto allow for detection without staining procedures.
26. Fibroblasts tend to concentrate on the borders of the gels, leading to artefacts on cancer cell Invasion in those areas. Pictures need to be taken in the central part of the gel.
27. Other analysis can also be performed. For example, Alternative Invasive Index (invading area/non-invading area), number of invading objects, area of invading cells (without measuring gel areas), etc.
28. NFs/CAFsFibroblasts can be allowed to remodel the gel up to 10 days before the cancer cells are seeded on top. The length of this step has to be determined empirically if other models are used.
29. The choice of selection depends on the resistance genes expressed by the cells (e.g., selection-based stable RNAi/overexpression systems previously inserted). We recommend determining the optimal final concentration beforehand.
30. Thorough washing with PBS and medium is important; otherwise cancer cells added subsequently may be affected. If this becomes problematic, we recommend generating stable cancer cells resistant to the selective compound.
